# Functional characterization of p53 pathway components in the ancient metazoan *Trichoplax adhaerens*

**DOI:** 10.1038/srep33972

**Published:** 2016-09-28

**Authors:** Jia Wei Siau, Cynthia R. Coffill, Weiyun Villien Zhang, Yaw Sing Tan, Juliane Hundt, David Lane, Chandra Verma, Farid Ghadessy

**Affiliations:** 1 p53Lab (A*STAR), 8A Biomedical Grove, #06-04/05 Neuros 138648, Singapore; 2 Bioinformatics Institute (A*STAR), 30 Biopolis Str, 07-01 Matrix 138671, Singapore; 3 Technical University Munich, School of Life Sciences Weihenstephan, Alte Akademie 8, 85354 Freising, Germany; 4 School of Biological Sciences, Nanyang Technological University, 60 Nanyang Drive 637551, Singapore; 5 Department of Biological Sciences, National University of Singapore, 14 Science Drive 4 117543, Singapore

## Abstract

The identification of genes encoding a p53 family member and an Mdm2 ortholog in the ancient placozoan *Trichoplax adhaerens* advocates for the evolutionary conservation of a pivotal stress-response pathway observed in all higher eukaryotes. Here, we recapitulate several key functionalities ascribed to this known interacting protein pair by analysis of the placozoan proteins (Tap53 and TaMdm2) using both *in vitro* and cellular assays. In addition to interacting with each other, the Tap53 and TaMdm2 proteins are also able to respectively bind human Mdm2 and p53, providing strong evidence for functional conservation. The key p53-degrading function of Mdm2 is also conserved in TaMdm2. Tap53 retained DNA binding associated with p53 transcription activation function. However, it lacked transactivation function in reporter genes assays using a heterologous cell line, suggesting a cofactor incompatibility. Overall, the data supports functional roles for TaMdm2 and Tap53, and further defines the p53 pathway as an evolutionary conserved fulcrum mediating cellular response to stress.

Genome sequencing of ancient organisms including the non-bilaterian sea anemone *Nematostella vectensis*, the fresh water cnidiarin *Hydra magnipapillata* and the placazoan *Trichoplax adhaerans* has revealed conservation of pathway genes involved in crucial cellular processes^1^,^2^,^3^. Significantly, genes related to p53 family members have been identified, and in certain organisms their function validated experimentally^4^,^5^. As “guardian of the genome”, the p53 tumour suppressor is a key determinant of cellular response to genotoxic insult^6^,^7^. In response to stress, p53-dependent transactivation of target genes can result in numerous outcomes ranging from cell cycle arrest to apoptosis. In the absence of stress, a negative feedback loop maintains low cellular levels of p53 through degradation mediated by Mdm2, the E3 ubiquitin ligase highly specific for p53^8^,^9^,^10^,^11^. Interventions that either reduce or remove Mdm2 function typically result in p53-mediated phenotypic outcomes. Notably, genetic deletion of Mdm2 is embryonically lethal in mouse models unless accompanied by co-deletion of p53, highlighting its indispensable role in higher eukaryote p53 regulation^12^,^13^. p53 and other family members (p63, p73) are also emerging as important players in development and cell differentiation processes^14^.

Recently, genes encoding a potential p53 family member and an Mdm2 ortholog were identified in the early metazoan *Trichoplax* *adhaerans* F. E. Schulze, arguably one of the simplest animals comprising only 5 cell types^4^,^15^,^16^,^17^. The p53-Mdm2 feedback loop may therefore have persisted over a billion years. Exposure of *Trichoplax* to small molecule inhibitors of the human p53-Mdm2 interaction leads to increased programed cell death, suggesting the presence of functional homologs^18^. Further studies to validate function are hindered by both the currently limited genetic tractability of *Trichoplax* and the paucity of antibody reagents. We have therefore employed heterologous expression in mammalian cells to further address the functionality of the *Trichoplax* p53 family member and *Mdm2* genes. The results show conservation of the DNA-binding function of the *Trichoplax* p53 family member (referred to as Tap53 throughout) and interaction with *Trichoplax* Mdm2 (TaMdm2) that can be inhibited using a peptidic antagonist of the human p53-Mdm2 interaction. Furthermore, TaMdm2 is able to guide degradation of Tap53 in a heterologous human cell line, indicating spectacular evolutionary conservation of the degradation pathway.

## Results

**Figure 1:**
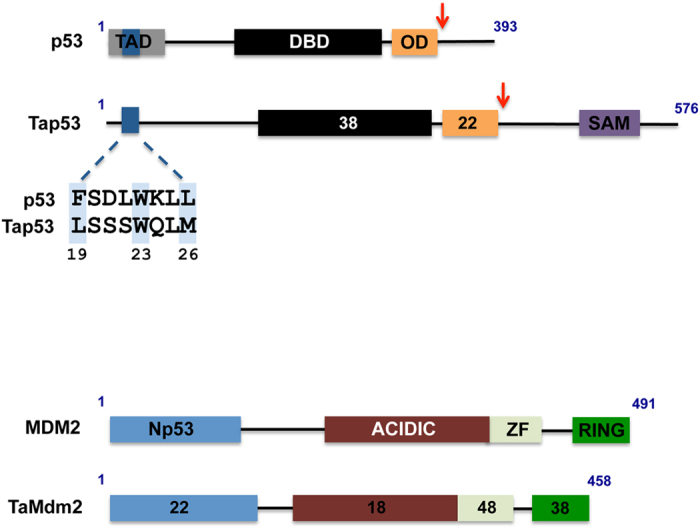
Domain structures of human and *Trichoplax* p53 and Mdm2 proteins. TAD: transcription activation domain; DBD: DNA binding domain; OD: oligomerisation domain; SAM: sterile alpha motif; Np53: N-terminal p53-interacting domain, ZF: zinc finger. Numbers within boxes indicate percentage amino acid sequence identity with p53 or MDM2. Dark blue box denotes N-terminal region of p53 interacting with MDM2 with sequence alignment shown expanded. Critical interacting residues in p53 and the corresponding residues in Tap53 are shaded light blue. Red arrows denote truncation points to generate p53Δ and Tap53Δ.

**Figure 2:**
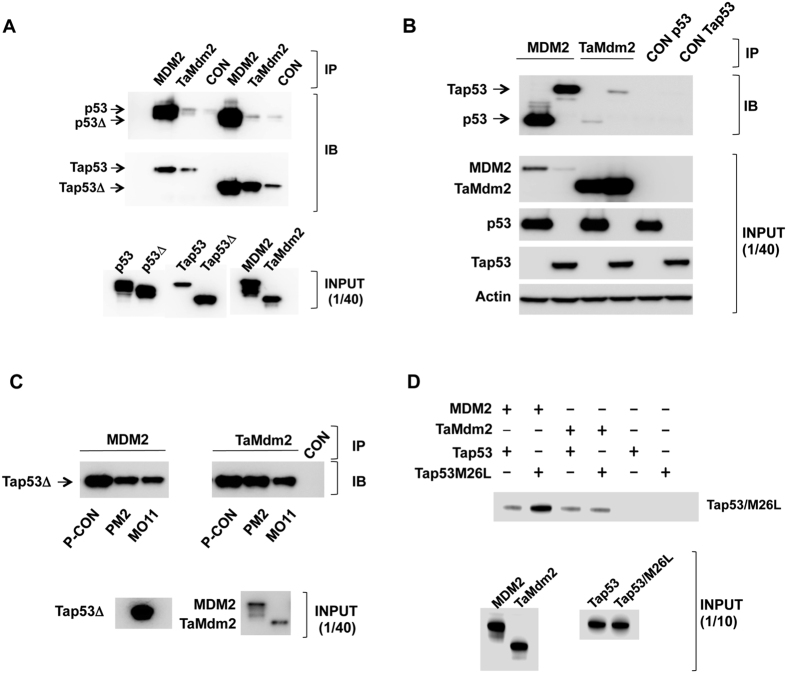
(**a**) Pull-down assay showing interaction of *in vitro* expressed TaMdm2 and Tap53. (**b**) Pull-down assay showing interaction of TaMdm2 and Tap53 expressed in H1299 cells. Tap53 detected using anti-FLAG antibody, p53 detected using DO1 antibody. (**c**) Disruption of the *in vitro* Tap53Δ-MDM2 interaction by the stapled peptide antagonists PM2 and MO11 (left panel). Disruption of Tap53Δ-TaMdm2 interaction by the stapled peptide antagonist MO11 (right panel). (**d)** Pull-down assay showing interactions of *in vitro* expressed Tap53 and Tap53M26L with MDM2 and TaMdm2. Control lanes (CON) show amounts of indicated p53 proteins pulled down in absence of MDM2/TaMdm2 immobilisation on beads. The calculated molecular weights of the indicated proteins are 56.3 KDa (MDM2, C-terminal HA tag), 43.7 KDa (p53), 39.5 KDa (p53Δ), 53 KDa (TaMdm2, C-terminal HA tag), 66.2 KDa (Tap53, C-terminal FLAG tag), 49.1 KDa (Tap53Δ, C-terminal FLAG tag). See Supplementary Information for uncropped blots.

We first carried out pull-down assays using proteins expressed *in vitro* to measure interactions. Both full-length and C-terminally truncated p53 variants (p53Δ, Tap53Δ) were used. p53 was truncated to remove the p53 C-terminal domain not critical for Mdm2 interaction and the corresponding truncation was made in Tap53 (Fig. 1). The results showed the expected strong interaction between human Mdm2 (referred to as MDM2 hereon) and both full length and C-terminally truncated p53. Similarly, TaMdm2 was seen to interact with Tap53 and Tap53Δ (Fig. 2a). Whilst the interaction with full-length Tap53 appears weaker, this is most likely due to its comparatively reduced expression levels. Notably, cross species interaction was observed, with MDM2 showing strong interaction with both full-length and truncated Tap53. TaMdm2 showed very weak interaction with p53 and p53Δ. Pull-down assays using proteins expressed in the p53-null H1299 cell line^19^ essentially recapitulated the *in vitro* data. TaMdm2 interacted with Tap53 and p53, albeit much weaker than for corresponding interactions of MDM2 (Fig. 2b). However, the amount of Tap53 pulled down is likely to be under-represented due to presence of competing endogenous MDM2 in H1299 cells.

The major interaction site of p53 with MDM2 resides in its N-terminal domain. This is defined by a contiguous sequence (amino acids 19–26) that comprises three signature amino acids (F19, W23 and L26) whose side chains interact with discrete pockets in a hydrophobic cleft of the MDM2 N-terminal domain (Fig. 1)^20^. Stapled peptide antagonists derived from this sequence have been shown to be potent inhibitors of the p53-MDM2 interaction^21^. Two stapled peptides, PM2 and MO11^22^ were both able to inhibit the interaction of MDM2 with Tap53Δ when compared to a negative control stapled peptide (P-CON)(Fig. 2c, left panel). In the case of the Tap53Δ-TaMdm2 interaction, only MO11 was able to inhibit the reaction (Fig. 2c, right panel). MO11 is a higher affinity derivative of PM2. As both these peptide antagonists compete with the N-terminal domain of p53 for binding to MDM2^23^, this indicates conservation of interaction of the N-terminal domain of Tap53 with TaMdm2. The prototypical small molecule MDM2 inhibitor Nutlin^24^ did not inhibit the *in vitro* Tap53Δ-TaMdm2 interaction (Fig. S1). This is not surprising as Nutlin binding can be compromised by very small changes in MDM2 structure^25^,^26^. However, homology modeling further highlights overall structural conservation between the MDM2 and TaMdm2 N-terminal domains and interactions with p53/Tap53 N-terminal interacting region (Fig. S2).

Of the three signature residues in the MDM2-interacting region of p53, only W23 is conserved in Tap53, with F19 and L26 respectively being replaced by L and M (Fig. 1). We therefore mutated the methionine in Tap53 to leucine, as this larger side chain is readily accommodated by MDM2^20^. The results show increased interaction of Tap53-M26L with MDM2 (Fig. 2d), further defining the key role of this region for MDM2 engagement. Interestingly, the interaction of this mutant with TaMdm2 was similar to wild-type. This may indicate plasticity in the N-terminal domain of TaMdm2 that accommodates the larger side chain and possibly greater dependence on a secondary p53-MDM2 interaction site^27^,^28^,^29^ that may be conserved in the Tap53-TaMdm2 interaction.

**Figure 3:**
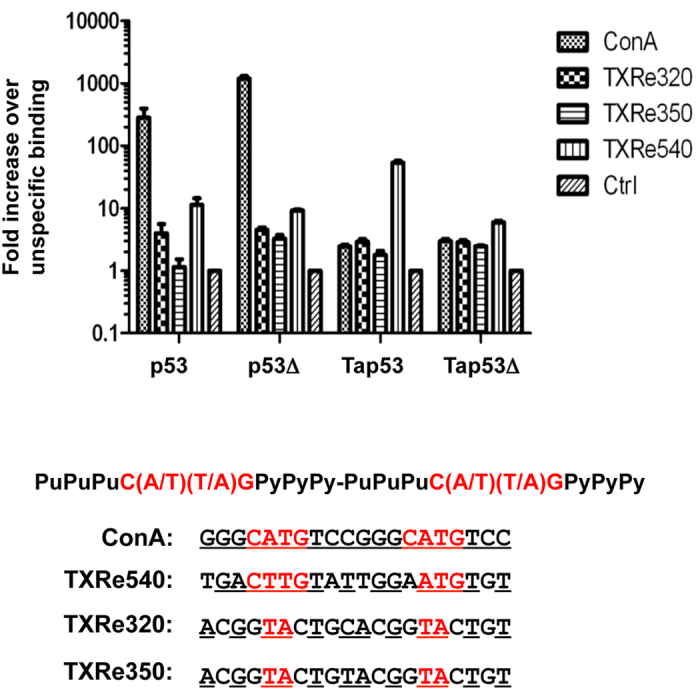
*In vitro* real-time PCR assay showing binding of indicated p53s to consensus (ConA) and three putative *Trichoplax* p53 response elements (TXRe540, TXRe320, TXRe350). Binding expressed as fold increase over binding to non-specific control DNA plotted on logarithmic scale. n = 2 ± SD. Shown also is consensus p53 motif comprising two 10bp half-sites separated by up to 13 nucleotides. Nucleotides corresponding to consensus are underlined and in red.

We next investigated the DNA binding function of Tap53 using a real-time PCR based assay^30^. In addition to probing with a consensus p53 response element (ConA)^31^, we used three sequences identified upstream of the TaMdm2 coding region (TXRe320, TXRe350 and TXRe540, respectively 320, 350 and 540bp upstream of coding region) as potential Tap53 binding sites (Fig. 3). As part of a negative feedback loop, MDM2 is a direct transcriptional target of p53. Hence these binding sites may represent evidence for the feedback loop in *Trichoplax*. The results show increased binding of both Tap53 and Tap53Δ to all response elements tested when compared to a control DNA sequence, with binding to TXRe540 being favoured (~30-fold increased binding over control DNA for Tap53)(Fig. 3). p53 also bound this sequence, albeit relatively weaker than the consensus sequence (respectively ~10 versus 120-fold binding over control DNA).

**Figure 4:**
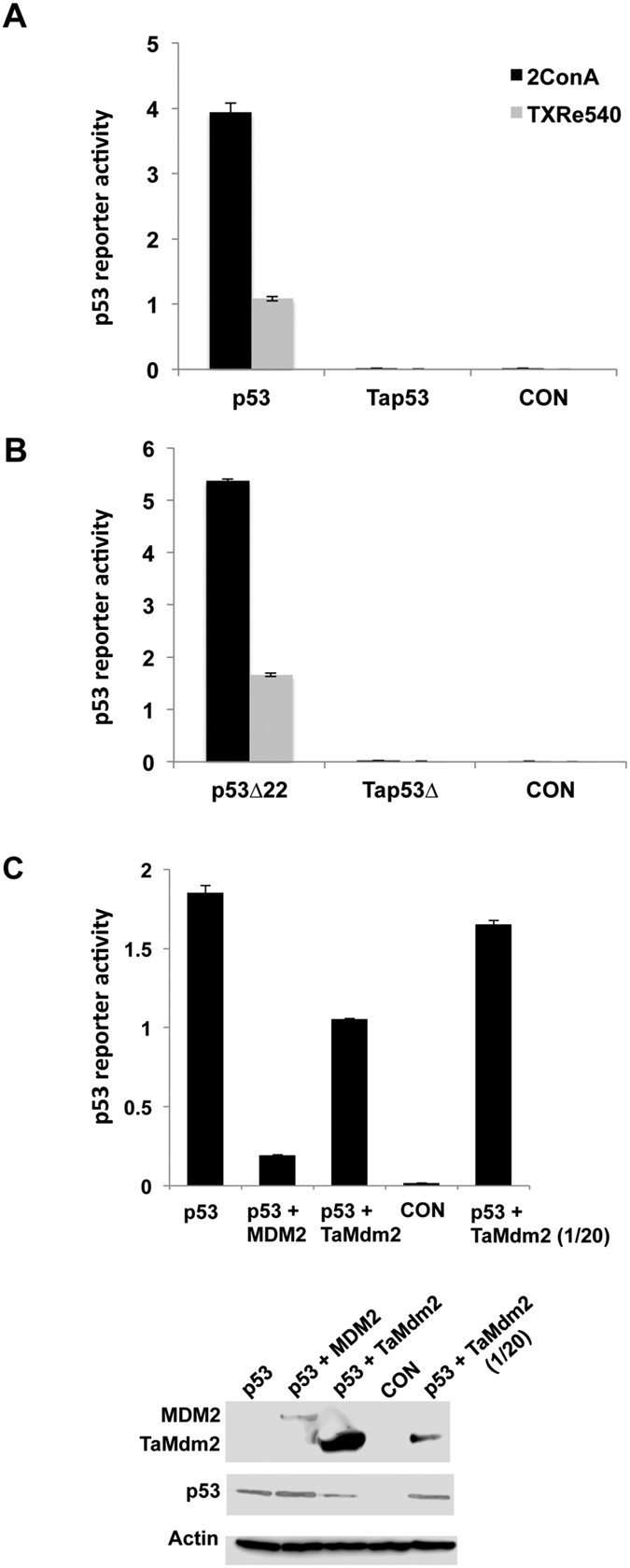
(**a,b**) Reporter gene assays using consensus (2ConA) and a putative *Trichoplax* p53 response element (TXRe540) show no transcriptional activation for Tap53 and Tap53Δ in H1299 cells. p53 is however capable of transactivation using TXRe540 response element. (**c**) Co-expression of TaMdm2 results in reduced p53 function in reporter gene assay using 2ConA promoter. TaMdm2 expression was also reduced (20-fold reduction of TaMdm2 expression plasmid co-transfected) to give a more comparable yield with MDM2 as shown in Western blot below. Data shows average of 2 independent experiments ± SD. See Supplementary Information for uncropped blots.

Tap53 was then assayed for transactivation function using p53 reporter gene assays in H1299 cells. In addition to using a reporter gene driven by tandem copies of the consensus p53 response element (2ConA), we also looked at the TXRe540 response element as Tap53 bound this preferentially (Fig. 3). Tap53 was unable to transactivate either reporter gene construct. In contrast, both p53 and p53Δ showed activity with both reporters. This was reduced ~4-fold for the TXRe540 reporter, in agreement with the comparatively weaker binding of p53 to the TXRe540 response element (Fig. 4a,b).

**Figure 5:**
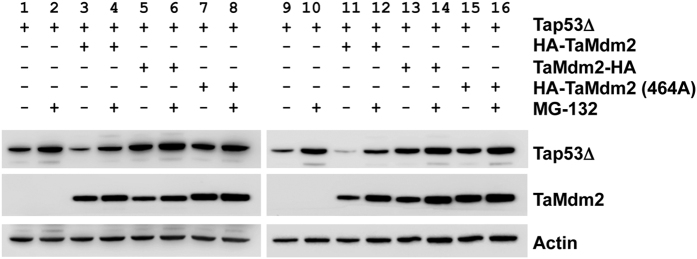
Degradation of Tap53Δ by TaMdm2 in DKO and TKO cells. Western blot of Tap53Δ levels following co-transfection with various TaMdm2 expressing constructs in either DKO (lanes 1–8) or TKO (lanes 9–16) cells. Lanes: (1 and 9) Tap53Δ; (2 and 10) Tap53Δ with MG132; (3 and 11) Tap53Δ + HA-TaMdm2; (4 and 12) Tap53Δ + HA-TaMdm2 with MG132; (5 and 13) Tap53Δ + TaMdm2-HA; (6 and 14) Tap53Δ + TaMdm2-HA MG132; (7 and 15) Tap53Δ + HA-TaMdm2(C464A); (8 and 16) Tap53Δ + HA-TaMdm2(C464A) with MG132. TaMdm2 levels can be seen in the middle panel and the loading control can be found in the lower panel. See Supplementary Information for uncropped blots.

MDM2 and TaMdm2 were next co-transfected with p53 into H1299 cells and p53 transactivation measured using the 2ConA reporter gene. MDM2 co-transfection led to the expected reduction of p53 function (Fig. 4c). TaMdm2 co-expression also reduced the activity of p53, albeit not as efficiently as MDM2 considering its very high expression level in this cell line (Fig. 4c). This is likely due in part to the weak interaction between p53 and TaMdm2 observed both in this cell line and *in vitro* (Fig. 2). To further address TaMdm2 cellular function, plasmids encoding TaMdm2 and Tap53 were co-transfected into the p53/Mdm2-null DKO cell line and the p53/Mdm2/Mdm4-null cell line TKO^13^. The TaMdm2 was tagged with the HA epitope at either the N- or C- terminus. The results showed that only N-terminally tagged TaMdm2 was able to reduce levels of co-expressed Tap53 (Fig. 5, compare lanes 3 and 5 or lanes 11 and 13). The ability of the proteasome inhibitor MG132 to block this degradation (compare lanes 3 and 4 or lanes 11 and 12) implies that, as with vertebrate Mdm2-p53, TaMdm2 is targeting Tap53 for proteasomal degradation. The inactivity of the C-terminally tagged TaMdm2 agrees with studies showing that MDM2 ubiquitination function can be inhibited by addition of tags/fusion partners adjacent to the C-terminal RING domain important for E3 ligase activity^32^. Likewise, mutation of a critical cysteine residue in the RING domain (equivalent to MDM2 C464) prevents the degradation of Tap53 (compare lanes 7 and 2 or lanes 15 and 11). These results indicate that ancient TaMdm2 interacts with mammalian E2 proteins and other components in the proteasomal pathway and emphasize the co-evolution of TaMdm2 and Tap53 in one of the earliest animal species.

**Figure 6:**
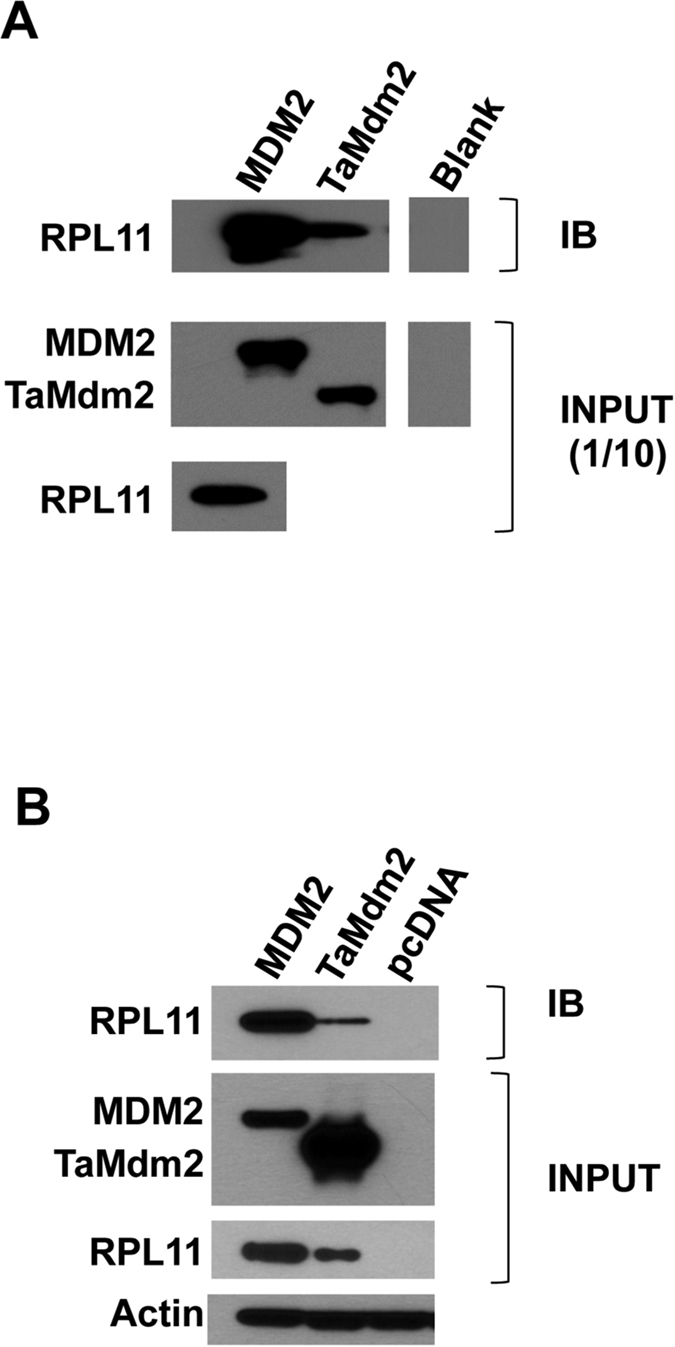
(**a**) Pull-down assay showing interaction of *in vitro* expressed TaMdm2 and RPL11. (**b**) Pull-down assay showing interaction of ectopically expressed TaMdm2 and RPL11 in H1299 cells. See Supplementary Information for uncropped blots.

The ribosomal protein component L11 has been shown to interact with MDM2 and regulate its function^33^,^34^. As with MDM2, TaMdm2 showed clear interaction with human L11 when measured by pull-down assay using *in vitro* expressed proteins (Fig. 6a). Similarly, both proteins were able to pull down human L11 in H1299 cells when expressed ectopically (Fig. 6b). The zinc finger domain of MDM2 has been shown to mediate interaction with L11^35^,^36^. This domain is the most highly conserved between the human and *Trichoplax* proteins (Fig. 1), again demonstrating strong evolutionary conservation of TaMdm2 function.

**Figure 7:**
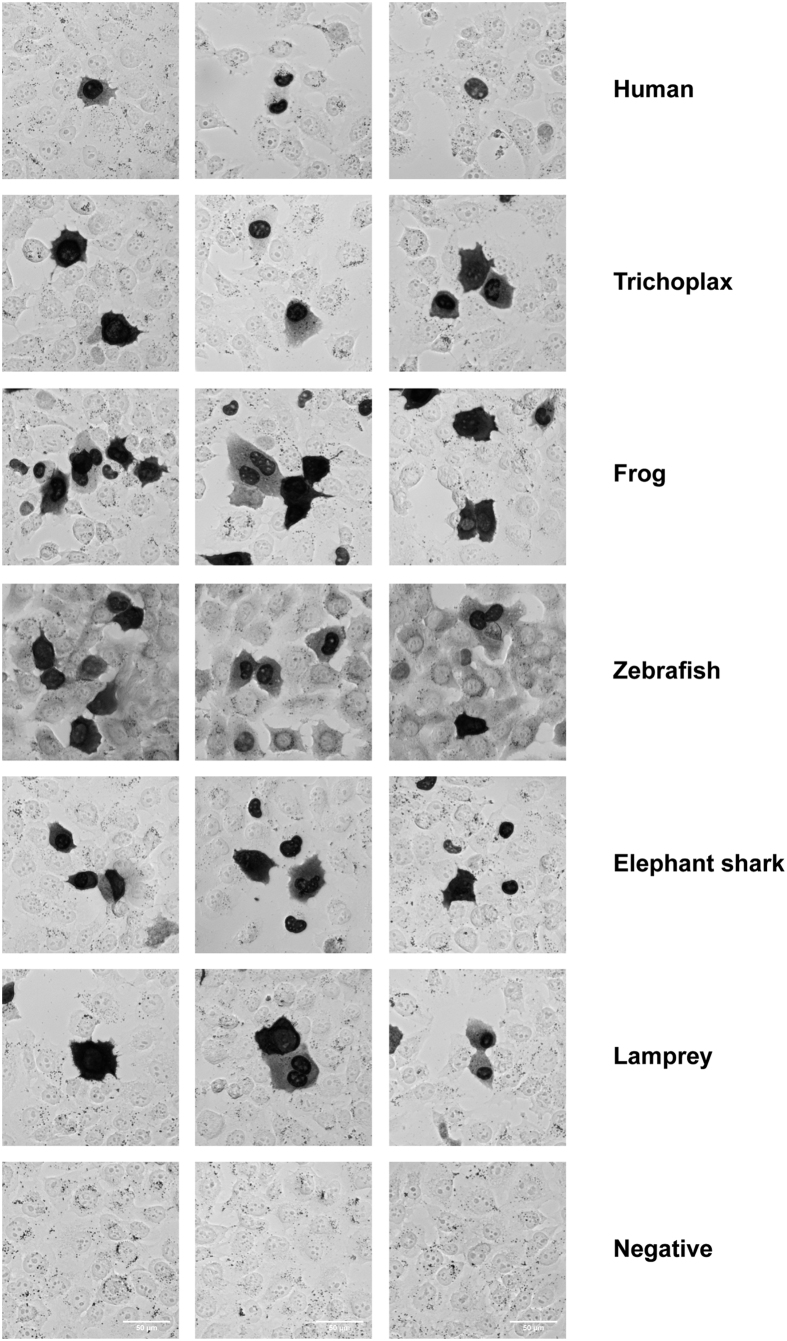
Immunocytochemical staining for p53 from divergent species. p53-null H1299 cells were transiently transfected with mammalian expression vectors encoding p53 from different species and probed with anti-p53 (DO1, human), anti-FLAG (trichoplax, elephant shark, lamprey and negative control), anti-HA (frog), anti-p53 (9.1, zebrafish) antibodies. The dark staining depicts p53 localization within each transfected cell (note that not all cells within the frame are transfected). The background staining was low for all antibodies except the hybridoma supernatant used for zebrafish p53 detection. Three representative images are shown for each species used to show the presence of nuclear and cytoplasmic staining.

As a transcription factor, human p53 is predominantly located in the nucleus. Sequence alignment of Tap53 shows conservation of positively charged residues in the region of the known C-terminal bipartite human p53 nuclear localization sequence (NLS)^4^,^37^. Also, there is strong conservation of a hydrophobic motif in the tetramerization domain comprising the nuclear export signal (NES)^38^. Immunocytochemistry analysis shows both nuclear and cytoplasmic distribution of Tap53 in H1299 cells, suggesting the presence of functional NLS and NES motifs (Fig. 7). Additional analysis of 4 other divergent species (frog, zebrafish, elephant shark, lamprey) also indicated similar p53 staining patterns.

## Discussion

The identification of p53-pathway components in the genome of *Trichoplax adhaerans* suggests exceptional evolutionary conservation in this early metazoan. We have recapitulated several of the core functions attributed to two of the key players in this pathway, a p53 family member and Mdm2. The p53 family member was shown to bind both a high affinity consensus p53 response element and a sequence identified in the promoter region of TaMdm2 with similarity to the canonical p53 response element. Human p53 was able to drive transactivation of a reporter gene using the *Trichoplax* response element. Mdm2 transcription in higher eukaryotes is regulated directly by p53 binding to response elements in its promoter. The functional sequences identified in *Trichoplax* therefore provide evidence towards conservation of the p53-Mdm2 feedback loop. Confirmation will require further studies in the native organism.

**Figure 8:**
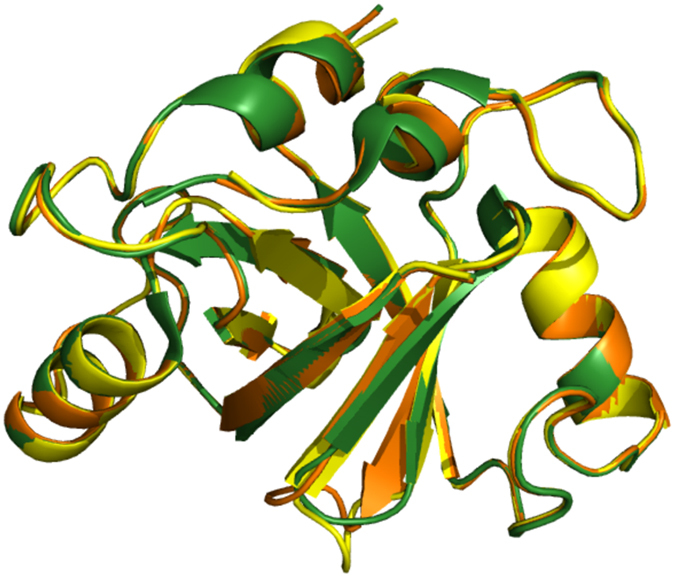
Homology models of MDM2–MDM4 RING domain heterodimer (green), TaMdm2 RING domain homodimer (yellow) and TaMdm2–mouse Mdm4 RING domain heterodimer (orange).

Optimal degradation of p53 by Mdm2 requires heterodimer formation via RING domain interaction with its paralog, Mdm4, in higher eukaryotes^39^,^40^,^41^,^42^,^43^. Both *Mdm2* and *Mdm4* likely arose from a gene duplication event more than 440 million years ago, prior to the emergence of vertebrates^44^,^45^. In common with other invertebrates including the sea squirt (*C. intestinalis*), acorn worm (*S. kowalevskii*), Florida lancelet (*B. floridae*), bay mussel (*M. trossulus*), and owl limpet (*L. gigantean*), only a single *Mdm* gene is evident in *Trichoplax*^44^,^46^. The product of this gene was able to degrade Tap53 in Mdm2/Mdm4-null TKO cells (Fig. 5), suggesting it can function independently. Homology modeling provides support for a TaMdm2 RING domain homodimer. This structure shows high similarity to a model of the TaMdm2–mouse Mdm4 RING domain heterodimer and the solved MDM2–MDM4 RING domain heterodimer structure (Fig. 8). The TaMdm2 homodimer is therefore likely to play similar functional roles in the degradation of p53, in agreement with the experimental results.

In TKO cells, the levels of Tap53 slightly decreased even in the absence of an Mdm2 protein and increased when co-expressed with catalytically inactive TaMdm2 variants (Fig. 5). This suggests the presence of another protein in TKO cells capable of targeting Tap53 for degradation. It is possible that binding of the TaMdm2 mutant to Tap53 shields it from degradation. Potential candidates include HUWE1 and COP1, ubiquitin ligases shown to target p53 independently of Mdm2/Mdm4^47^,^48^ with homologues present in the *Trichoplax* genome.

The p53 family member identified in *Trichoplax* was not capable of significant reporter gene activation in a human cell line, despite translocation to the nucleus. Compared to other domains, the N-terminal transactivation domain of this family member shows the least homology with human p53. This would suggest an inability to interact with basal transcription factors that are recruited by p53. Also, there may be subtle contextual variation between human and *Trichoplax* p53 response elements that is not reflected in the human p53 reporter gene assay employed. As in *Trichoplax*, p53 family members identified in other divergent species such as the sea lamprey and mussel do not show transactivation in heterologous cells^45^,^46^.

Our results provide increasing evidence for the presence of a single functional p53 family member and an independently competent Mdm2 in *Trichoplax adhaerens.* The minimised p53 pathway set in this simple animal further validates its use as a reductionist model organism to study development and cancer.

## Methods

Unless otherwise specified, all oligonucleotides and codon-optimised genes (derived from UniProt sequences B3RT05 and B3RZS6) were from Integrated DNA Technologies (Singapore), restriction enzymes from NEB and chemical reagents from Sigma.

### Oligonucleotides

INF-T05-ndeF5′-AAGGAGATATACATATG GCGAGCTGCGATAGCGCGACC -3′

INF-T05-HA-bamR5′-GCTCGAATTCGGATCCTCATTACGCATAATCCGGCACATCATACGGATAGCCAATAAAGTTCTGCACAATCAGTTCAAT -3′

INF-ZS6-ndeF5′-AAGGAGATATACATATG AGCGATGAACCGACCCTGAGC -3′

INF-ZS6-FLAG-bamR5′-GCTCGAATTCGGATCCTCATTTATCATCATCATCTTTATAATCCAGGGTAATGGTCTGGCGCAGGGTAAAGCG -3′

INF-ZS6delta-FLAG-bamR5′- GCTCGAATTCGGATCCTCA TTTATCATCATCATCTTTATAATC TTC GTT AAT CTG CGC ATC GCT CAG -3′

INFLAGL11-F5′- AAGGAGATATACATATGGATTATAAAGATGATGATGATAAAGCGCAGGATCAAGGTGAAAAGG -3′

INFL11-R5′- ATTCCGATATCCATGGTTATTTGCCAGGAAGGATGATCC -3′

petF25′- CATCGGTGATGTCGGCGAT -3′

petR5′- CGGATATAGTTCCTCCTTTCAGCA -3′

jwConA-petF25′- GAC ACG GGC ATG TCC GGG CAT GTC CGG GCA CAT CGG TGA TGT CGG CGA T -3′

TXRe-540-F25′- TTC TGA TTG ACT TGT ATT GGA ATG TGT ACA TCG GTG ATG TCG GCG ATA T -3′

TXRe-320-F25′- CAT ACC GTA CAG TAC CGT GCA GTA CCG TAC ATC GGT GAT GTC GGC GAT AT -3′

TXRe-350-F25′- TAC CGT ACA GTA CCG TAC AGT ACC GTC ATC GGT GAT GTC GGC GAT AT -3′

petF35′- ATAGGCGCCAGCAACCGCACCTG -3′

WpetR15′- TAATTTCGCGGGATCGAGATCT -3′

INF-T05-Fv25′- CAGTGTGGTGGAATTCGCCACCATGGCCAGCTGCGACAGCGCCACC -3′

INF-T05-Rv25′- GAAGGGCCCTCTAGATCAGGCGTAGTCGGGCACGTCGTAGGGGTAGCCGATGAAGTTCTGCACGATCAGCTCGAT -3′

INF-ZS6-Fv25′- CAGTGTGGTGGAATTCGCCACCATGAGCGACGAGCCCACCCTGAGC –3′

INF-ZS6-Rv25′- GAAGGGCCCTCTAGATCACTTGTCGTCGTCGTCCTTGTAGTCCAGGGTGATGGTCTGTCTCAGGGTGAATCT -3′

INF-ZS6delta-Rv25′-GAAGGGCCCTCTAGATCA CTTGTCGTCGTCGTCCTTGTAGTCCTCGTTGATCTGGGCGTCGCTCAG -3′

TXRe-540-petF45′- TTC TGA TTG ACT TGT ATT GGA ATG TGT A GATGCGTCCGGCGTAGAGGATCG -3′

Bgal-Rev5′- GGCCGCCACCGCGGTGGAG - 3′

Txp53-M26L-QC15′- AGCAGCTGGCAGCTGcTGATTGATGAAATTA -3′

Txp53-M26L-QC25′- TAATTTCATCAATCAgCAGCTGCCAGCTGCT -3′

Txp53-M-M26L-QC15′- AGCAGCTGGCAGCTGcTGATCGACGAGATCA -3′

Txp53-M-M26L-QC25- TGATCTCGTCGATCAgCAGCTGCCAGCTGCT -3′

### Vector construction

TaMdm2-HA-PET22b and Tap53-FLAG-PET22b – plasmids encoding for Ta Mdm2 and Tap53 respectively, were generated via amplification of pUC57-T05 and pUC57-ZS6 with primers INF-T05-ndeF and INF-T05-HA-bamR, and INF-ZS6-ndeF and INF-ZS6-FLAG-bamR followed by ligation to pET22b via NdeI and BamHI sites. Infusion cloning (Clontech) was carried out on pUC57-ZS6 with primers INF-ZS6-ndeF and INF-ZS6delta-FLAG-bamR to create Tap53∆-FLAG-pET22b. Infusion cloning was carried out on FLAG-L11-pCDNA with primers INFLAGL11-F and INFL11-R to create FLAG-L11-pET22. Constructs were amplified with petF2 and petR to generate amplicons with T7 promoter and ribosome binding site required for *in vitro* transcription-translation (IVT).

Amplification of pET22b with either jwConA-petF2, TXRe-540-F2, TXRe-320-F2 and TXRe-350-F2 with petR was carried out to create short DNA fragments with response elements ConA, TXRe530, TXRe320 and TXRe350 appended at the N-terminus. Control DNA (minus response element) was created by using petF2 and petR to amplify pET22b. Purified DNA was used for DNA binding assay (described below).

For cell culture work, INF-T05-Fv2 and INF-T05-Rv2 were used on pUC57-M-T05 to generate insert for infusion cloning of TaMdm2 with a C-terminal HA tag into pCDNA3.1a(+) to yield TaMdm2-HA-pcDNA. Infusion cloning of Tap53 with a C-terminal FLAG tag was also carried out on pUC57-M-ZS6 with primers INF-ZS6-Fv2 and INF-ZS6-Rv2 to create Tap53-FLAG-pcDNA. The C-terminus truncated Tap53∆-FLAG-pcDNA was created using INF-ZS6delta-Rv2 as the reverse primer before infusion cloning was performed. The LacZ reporter plasmid TXRe540-Bgal-pcDNA was created using primers TXRe-540-petF4 and Bgal-rev on 2ConA-Bgal-pcDNA. Primers Txp53-M26L-QC1 and Txp53-M26L-QC2 was used on Tap53-FLAG-PET22b to introduce the mutation M26L for IVT work. The same mutation was introduced into Tap53-FLAG-pcDNA using primers Txp53-M-M26L-QC1 and Txp53-M-M26L-QC2 to create the mammalian vector.

### Immunoprecipitation of *in vitro*-expressed proteins and western blot analysis

Protein G beads were incubated with anti-HA antibody (1 μg per 10 μL beads) for 1 hour in PBST-3%BSA and subsequently washed twice in PBST-0.1%BSA. IVT-expressed MDM2/TaMdm2 was incubated with the beads on a rotator for 30 mins and removed. Stapled peptides were added at indicated concentrations and incubation carried out for 45 mins before removal. IVT-expressed p53/Tap53/L11 was then added to the mixture and incubation allowed for 45 mins. Beads were finally washed thrice in PBST-0.1% BSA and thrice with PBS, and bound proteins eluted by resuspension in 20 μL SDS-PAGE loading buffer and incubation at 95 °C for 5 minutes. Both the eluates and inputs were subjected to electrophoresis, transferred to nitrocellulose membranes and probed for p53 with horseradish peroxidase conjugated DO1 antibody (Santa Cruz), for Tap53/L11 with horseradish peroxidase conjugated anti-FLAG, or for MDM2/TaMdm2 with anti-HA antibody or followed by rabbit anti-mouse (Dakocytomation). The western blots were identified by Clarity™ ECL western blotting substrate (Bio-Rad).

### DNA binding assay and real-time PCR

Protein G beads (5 μL) (Invitrogen) were incubated with anti-FLAG or DO1 antibody (1 μg per 10μL beads) for 1 hour in PBST-3% BSA and subsequently washed twice in PBST-0.1% BSA. A 10 μL mixture comprising 10–20 nM IVT-expressed p53/Tap53 (3 μL *in vitro* expression mix added), 60 nM DNA fragments (containing ConA, TXRe320, TXRe350, TXRe540 response elements or control DNA) and water was prepared prior to incubation with the beads on a rotator for 1 hour at RT. Beads were finally washed thrice in PBST-0.1% BSA and thrice with PBS, and bound DNA eluted by resuspension in 20μL nuclease-free water and incubation at 95 °C for 5 mins. Real-time PCR quantifications of the eluates were performed using 250nM each of primers petF3 and WpetR1 using iQTM SYBR^®^ Green Supermix (Bio-Rad Laboratories) and quantified via CFX96 Real-Time System CCD camera (Bio-Rad Laboratories). Data was interpreted as fold differences (calculated based on cycle threshold differences) over non-specific DNA binding control (control DNA).

### Cell culture

H1299 p53^−/−^ cells were maintained in Dulbecco’s modified Eagle’s medium (DMEM) with 10% (v/v) foetal calf serum (FCS) and 1% (v/v) penicillin/streptomycin. The cells were seeded at 1.4 × 10^5^ cells/well in 6-well plates, 24 hours prior to transfection. Cells were co-transfected with different combinations of MDM2/TaMdm2/p53/Tap53 plasmid using Lipofectamine 2000 (Invitrogen) according to the manufacturer’s instructions. For β-galactosidase assay, 2ConA/TXRe540 LacZ reporter plasmid and luciferase transfection efficiency plasmid were also included during transfection. In all cases, the total amount of plasmid DNA transfected per well was equilibrated by addition of the parental vector pcDNA3.1a(+). Mouse embryonic fibroblast p53/Mdm2 double-knockout (DKO) and p53/Mdm2/Mdm4 triple-knockout (TKO) cells (kind gifts from Guillermina Lozano) were maintained in Dulbecco’s modified Eagle’s medium (DMEM) with 10% (v/v) foetal calf serum (FCS) and 1% (v/v) penicillin/streptomycin. To ensure a high transfection efficiency for reproducible amounts of p53 protein, DKO and TKO cells were co-transfected with Tap53Δ, TaMdm2 and vector plasmids using Nucleofector^®^ II (Lonza, Singapore) according to the manufacturer’s instructions for mouse embryonic fibroblasts with MEF2 Nucleofector solution and program A-023. In all cases, the total amount of plasmid DNA transfected per well was equalized by addition of parental vector. Where required, four hours prior to harvesting, cells were treated with proteasome inhibitor MG132 (Calbiochem/Merck/Millipore, Singapore).

### Immunoprecipitation of cell lysates and western blot analysis

H1299 p53^−/−^ cells were harvested 24 hours after transfection with modified RIPA buffer (50mM Tris-HCl (pH 7.4–8.0), 150mM NaCl, 1% NP-40) supplemented with both protease and phosphatase inhibitors. 10 μL of anti-HA antibody-coated protein G Dynabeads was used per reaction. Beads were washed twice with PBST-0.1% BSA and incubated with 200 μg of cell lysate on a rotator at 4 °C overnight before washing thrice in PBST-0.1% BSA and thrice with PBS, and bound proteins eluted by resuspension in 20 μL SDS-PAGE loading buffer and incubation at 95 °C for 5 minutes. Immunoblotting was carried out with the relevant antibodies and identified by Clarity™ ECL Western Blotting Substrate). 5 μg of H1299 p53^−/−^ cell lysate per reaction was also used to check expression levels of relevant proteins via western blot. DKO/TKO cells were harvested 24 hours after transfection by lysis with 95 °C SDS buffer (20mM Tris pH 8, 2% SDS and 10% glycerol) and sonicated. Following protein concentration determination by BCA Protein Assay (Thermo Scientific Pierce, Singapore), equal protein amounts of sample were added to LDS-PAGE loading buffer and incubated at 95 °C for 5 minutes. The proteins were then separated by SDS-PAGE and transferred to nitrocellulose. Immunoblotting was carried out with the relevant antibodies (anti-FLAG for Tap53Δ, anti-HA for TaMdm2 and anti-β-actin for loading control).

### β-Galactosidase assay

H1299 p53^−/−^ cells were harvested 24hours after transfection and β-galactosidase activities were assessed using the Dual-light System (Applied Biosystems) according to the manufacturer’s protocol. The β-galactosidase activity was normalized with luciferase activity for each sample.

### Immunocytochemistry and image acquisition

H1299 cells were grown on chambered coverglass slides (Lab-Tek; Thermo Fisher Scientific) for one day and then transfected using Turbofect^TM^ (Thermo Fisher Scientific) and constructs expressing p53 from various species. Mammalian expression constructs used were: pcDNA3.1-Hsp53 (human, accession number P04637); pcDNA3.1-FLAG-Tap53Δ (*Trichoplax adhaerens*, B3RZS6); pXJ40-HA-Xlp53 (frog, *Xenopus laevis*, P07193); pCS2+-HIS-Drp53 (zebrafish, *Danio rerio*, U60804); pCIneo-FLAG-Cmp53 (elephant shark, *Callorhinchus milii*, G9J1L8); and pCIneo-FLAG-Ljp53 (*Lamprey japonica*, KT960978). 24 hours following transfection the cells were washed with PBS, fixed for 10 min in 4% paraformaldehyde, washed in PBS, permeabilized using PBS + 0.5% Triton X-100 and then blocked in DMEM + 10% BSA. Cells were incubated with primary antibodies in blocking solution overnight at 4 °C, washed in PBS, probed with secondary anti-mouse HRP (Dako) antibodies for 45 min at RT, washed in PBS and then stained with diaminobenzidine (DAB; Dako) for 2 min. Primary antibodies used were: anti-p53 (DO1, Moravian Biotechnology Ltd), anti-HA (Santa Cruz), anti-p53 (anti-p53(9.1) anti-zebrafish p53 hybridoma supernatant), or anti-FLAG (Moravian Biotechnology Ltd). Untagged cDNA for Xlp53 was a kind gift from Julian Gannon and Vincenzo Costanzo while untagged cDNA for Cmp53 was generously supplied by Alison Lee and Byrappa Venkatesh.

Images were acquired on an inverted microscope (Olympus IX83) using bright field with an LUCPLFLN-40 × PH, NA 0.60 objective and a camera (Hamamatsu ORCA - Flash 4.0). Images were acquired at a resolution of 2048 × 2048 pixels, 16-bit grey scale and exported as TIFF using cellSens Dimension software (version 1.15). Images were cropped to 1200 × 1200 pixels and scale bar was added in Fiji ImageJ2 and then reduced in size for figure assembly using Illustrator (CS5; Adobe).

## Additional Information

**How to cite this article**: Siau, J. W. *et al*. Functional characterization of p53 pathway components in the ancient metazoan *Trichoplax adhaerens*. *Sci. Rep.* **6**, 33972; doi: 10.1038/srep33972 (2016).

## Supplementary Material

Supplementary Information

## References

[b1] Srivastava M. . The Trichoplax genome and the nature of placozoans. Nature 454, 955–960 (2008). 18719581 10.1038/nature07191

[b2] Putnam N. H. . Sea anemone genome reveals ancestral eumetazoan gene repertoire and genomic organization. Science 317, 86–94 (2007). 17615350 10.1126/science.1139158

[b3] Chapman J. A. . The dynamic genome of Hydra. Nature 464, 592–596 (2010). 20228792 10.1038/nature08830 PMC4479502

[b4] Lane D. P. . Mdm2 and p53 are highly conserved from placozoans to man. Cell Cycle 9, 540–547 (2010). 20081368 10.4161/cc.9.3.10516

[b5] Pankow S. & Bamberger C. The p53 tumor suppressor-like protein nvp63 mediates selective germ cell death in the sea anemone Nematostella vectensis. PLoS One 2, e782 (2007). 17848985 10.1371/journal.pone.0000782 PMC1964547

[b6] Kastan M. B., Onyekwere O., Sidransky D., Vogelstein B. & Craig R. W. Participation of p53 protein in the cellular response to DNA damage. Cancer Res 51, 6304–6311 (1991). 1933891

[b7] Kuerbitz S. J., Plunkett B. S., Walsh W. V. & Kastan M. B. Wild-type p53 is a cell cycle checkpoint determinant following irradiation. Proc Natl Acad Sci USA 89, 7491–7495 (1992). 1323840 10.1073/pnas.89.16.7491 PMC49736

[b8] Momand J., Zambetti G. P., Olson D. C., George D. & Levine A. J. The mdm-2 oncogene product forms a complex with the p53 protein and inhibits p53-mediated transactivation. Cell 69, 1237–1245 (1992). 1535557 10.1016/0092-8674(92)90644-r

[b9] Haupt Y., Maya R., Kazaz A. & Oren M. Mdm2 promotes the rapid degradation of p53. Nature 387, 296–299 (1997). 9153395 10.1038/387296a0

[b10] Kubbutat M. H., Jones S. N. & Vousden K. H. Regulation of p53 stability by Mdm2. Nature 387, 299–303 (1997). 9153396 10.1038/387299a0

[b11] Honda R., Tanaka H. & Yasuda H. Oncoprotein MDM2 is a ubiquitin ligase E3 for tumor suppressor p53. FEBS Lett 420, 25–27 (1997). 9450543 10.1016/s0014-5793(97)01480-4

[b12] Jones S. N., Roe A. E., Donehower L. A. & Bradley A. Rescue of embryonic lethality in Mdm2-deficient mice by absence of p53. Nature 378, 206–208 (1995). 7477327 10.1038/378206a0

[b13] Montes de Oca Luna R., Wagner D. S. & Lozano G. Rescue of early embryonic lethality in mdm2-deficient mice by deletion of p53. Nature 378, 203–206 (1995). 7477326 10.1038/378203a0

[b14] Molchadsky A., Rivlin N., Brosh R., Rotter V. & Sarig R. p53 is balancing development, differentiation and de-differentiation to assure cancer prevention. Carcinogenesis 31, 1501–1508 (2010). 20504879 10.1093/carcin/bgq101

[b15] Schierwater B. My favorite animal, Trichoplax adhaerens. Bioessays 27, 1294–1302 (2005). 16299758 10.1002/bies.20320

[b16] Guidi L., Eitel M., Cesarini E., Schierwater B. & Balsamo M. Ultrastructural analyses support different morphological lineages in the phylum Placozoa Grell, 1971. J Morphol 272, 371–378 (2011). 21246596 10.1002/jmor.10922

[b17] Schulze F. E. Trichoplax adhaerens, nov. gen., nov. spec. Zool. Anz. 6, 92–97 (1883).

[b18] von der Chevallerie K., Rolfes S. & Schierwater B. Inhibitors of the p53-Mdm2 interaction increase programmed cell death and produce abnormal phenotypes in the placozoon Trichoplax adhaerens (F.E. Schulze). Dev Genes Evol 224, 79–85 (2014). 24522962 10.1007/s00427-014-0465-0

[b19] Takahashi T. . Wild-type but not mutant p53 suppresses the growth of human lung cancer cells bearing multiple genetic lesions. Cancer Res 52, 2340–2343 (1992). 1559236

[b20] Kussie P. . Structure of the MDM2 oncoprotein bound to the p53 tumor suppressor transactivation domain. Science 274, 948–953 (1996). 8875929 10.1126/science.274.5289.948

[b21] Baek S. . Structure of the stapled p53 peptide bound to Mdm2. J Am Chem Soc 134, 103–106 (2012). 22148351 10.1021/ja2090367

[b22] Brown C. J. . Stapled peptides with improved potency and specificity that activate p53. ACS Chem Biol 8, 506–512 (2013). 23214419 10.1021/cb3005148

[b23] Chee S. M. . Structure of a stapled peptide antagonist bound to nutlin-resistant Mdm2. PLoS One 9, e104914 (2014). 25115702 10.1371/journal.pone.0104914 PMC4130638

[b24] Vassilev L. . *In vivo* activation of the p53 pathway by small-molecule antagonists of MDM2. Science 303, 844–848 (2004). 14704432 10.1126/science.1092472

[b25] Popowicz G. M. . Molecular basis for the inhibition of p53 by Mdmx. Cell Cycle 6, 2386–2392 (2007). 17938582 10.4161/cc.6.19.4740

[b26] Wei S. J. . *In vitro* selection of mutant HDM2 resistant to Nutlin inhibition. PLoS One 8, e62564 (2013). 23653682 10.1371/journal.pone.0062564 PMC3641235

[b27] Ma J. . A second p53 binding site in the central domain of Mdm2 is essential for p53 ubiquitination. Biochemistry 45, 9238–9245 (2006). 16866370 10.1021/bi060661u

[b28] Yu G. W. . The central region of HDM2 provides a second binding site for p53. Proc Natl Acad Sci USA 103, 1227–1232 (2006). 16432196 10.1073/pnas.0510343103 PMC1360574

[b29] Shimizu H. . The conformationally flexible S9-S10 linker region in the core domain of p53 contains a novel MDM2 binding site whose mutation increases ubiquitination of p53 *in vivo*. J Biol Chem 277, 28446–28458 (2002). 11925449 10.1074/jbc.M202296200

[b30] Goh W., Lane D. & Ghadessy F. Development of a novel multiplex *in vitro* binding assay to profile p53-DNA interactions. Cell Cycle 9, 3030–3038 (2010). 20714218 10.4161/cc.9.15.12436

[b31] Noureddine M. A. . Probing the functional impact of sequence variation on p53-DNA interactions using a novel microsphere assay for protein-DNA binding with human cell extracts. PLoS Genet 5, e1000462 (2009). 19424414 10.1371/journal.pgen.1000462 PMC2667269

[b32] Dolezelova P., Cetkovska K., Vousden K. H. & Uldrijan S. Mutational analysis of Mdm2 C-terminal tail suggests an evolutionarily conserved role of its length in Mdm2 activity toward p53 and indicates structural differences between Mdm2 homodimers and Mdm2/MdmX heterodimers. Cell Cycle 11, 953–962 (2012). 22333590 10.4161/cc.11.5.19445 PMC3323797

[b33] Lohrum M. A., Ludwig R. L., Kubbutat M. H., Hanlon M. & Vousden K. H. Regulation of HDM2 activity by the ribosomal protein L11. Cancer Cell 3, 577–587 (2003). 12842086 10.1016/s1535-6108(03)00134-x

[b34] Zhang Y. . Ribosomal protein L11 negatively regulates oncoprotein MDM2 and mediates a p53-dependent ribosomal-stress checkpoint pathway. Mol Cell Biol 23, 8902–8912 (2003). 14612427 10.1128/MCB.23.23.8902-8912.2003 PMC262682

[b35] Zhang Q., Xiao H., Chai S. C., Hoang Q. Q. & Lu H. Hydrophilic residues are crucial for ribosomal protein L11 (RPL11) interaction with zinc finger domain of MDM2 and p53 protein activation. J Biol Chem 286, 38264–38274 (2011). 21903592 10.1074/jbc.M111.277012 PMC3207392

[b36] Lindstrom M. S., Jin A., Deisenroth C., White Wolf G. & Zhang Y. Cancer-associated mutations in the MDM2 zinc finger domain disrupt ribosomal protein interaction and attenuate MDM2-induced p53 degradation. Mol Cell Biol 27 (2007). 10.1128/MCB.01307-06 PMC1800693 17116689

[b37] Liang S. H. & Clarke M. F. A bipartite nuclear localization signal is required for p53 nuclear import regulated by a carboxyl-terminal domain. J Biol Chem 274, 32699–32703 (1999). 10551826 10.1074/jbc.274.46.32699

[b38] Stommel J. M. . A leucine-rich nuclear export signal in the p53 tetramerization domain: regulation of subcellular localization and p53 activity by NES masking. EMBO J 18, 1660–1672 (1999). 10075936 10.1093/emboj/18.6.1660 PMC1171253

[b39] Marine J. C. . Keeping p53 in check: essential and synergistic functions of Mdm2 and Mdm4. Cell Death Differ 13, 927–934 (2006). 16543935 10.1038/sj.cdd.4401912

[b40] Kawai H., Lopez-Pajares V., Kim M. M., Wiederschain D. & Yuan Z. M. RING domain-mediated interaction is a requirement for MDM2′s E3 ligase activity. Cancer Res 67, 6026–6030 (2007). 17616658 10.1158/0008-5472.CAN-07-1313

[b41] Linke K. . Structure of the MDM2/MDMX RING domain heterodimer reveals dimerization is required for their ubiquitylation in trans. Cell Death Differ 15, 841–848 (2008). 18219319 10.1038/sj.cdd.4402309

[b42] Pant V., Xiong S., Iwakuma T., Quintas-Cardama A. & Lozano G. Heterodimerization of Mdm2 and Mdm4 is critical for regulating p53 activity during embryogenesis but dispensable for p53 and Mdm2 stability. Proc Natl Acad Sci USA 108, 11995–12000 (2011). 21730132 10.1073/pnas.1102241108 PMC3141986

[b43] Huang L. . The p53 inhibitors MDM2/MDMX complex is required for control of p53 activity *in vivo*. Proc Natl Acad Sci USA 108, 12001–12006 (2011). 21730163 10.1073/pnas.1102309108 PMC3141917

[b44] Momand J., Villegas A. & Belyi V. A. The evolution of MDM2 family genes. Gene 486, 23–30 (2011). 21762762 10.1016/j.gene.2011.06.030 PMC3162079

[b45] Coffill C. R. . The p53-Mdm2 interaction and the E3 ligase activity of Mdm2/Mdm4 are conserved from lampreys to humans. Genes Dev 30, 281–292 (2016). 26798135 10.1101/gad.274118.115 PMC4743058

[b46] Muttray A. F., O’Toole T. F., Morrill W., Van Beneden R. J. & Baldwin S. A. An invertebrate mdm homolog interacts with p53 and is differentially expressed together with p53 and ras in neoplastic Mytilus trossulus haemocytes. Comp Biochem Physiol B Biochem Mol Biol 156, 298–308 (2010). 20417299 10.1016/j.cbpb.2010.04.008 PMC2916963

[b47] Chen D. . ARF-BP1/Mule is a critical mediator of the ARF tumor suppressor. Cell 121, 1071–1083 (2005). 15989956 10.1016/j.cell.2005.03.037

[b48] Dornan D. . The ubiquitin ligase COP1 is a critical negative regulator of p53. Nature 429 (2004). 10.1038/nature02514 15103385

