# Supplementary material for: Functional characterization of p53 pathway components in the ancient metazoan Trichoplax adhaerens
Source: Sci Rep. 2016 Sep 28;6:33972. doi: 10.1038/srep33972 (PMC5039725; doi:10.1038/srep33972)
Supplement: Supplementary Information [file srep33972-s1.pdf]

Supplementary Information

Functional characterization of p53 pathway components in the ancient metazoan *Trichoplax adhaerens.*

Jia Wei Siau, Cynthia R. Coffill, Weiyun Villien Zhang, Yaw Sing Tan, Juliane Hundt, David Lane, Chandra Verma, Farid Ghadessy

Figure S1

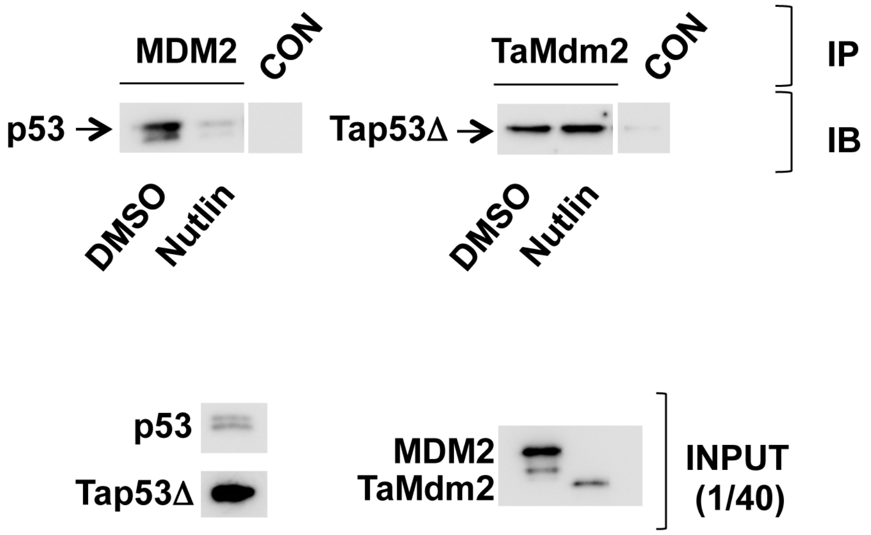

Figure S1. Pull-down assay showing interactions of *in vitro* expressed Tap53Δ/ TaMdm2 and p53/HDM2. Nutlin (10μM) is able to disrupt the p53/HDM2 interaction (left) but not the Tap53Δ/TaMdm2 interaction (right). Control lanes (CON) show amounts of indicated p53 proteins pulled down in absence of MDM2/TaMdm2 immobilisation on beads

Figure S2

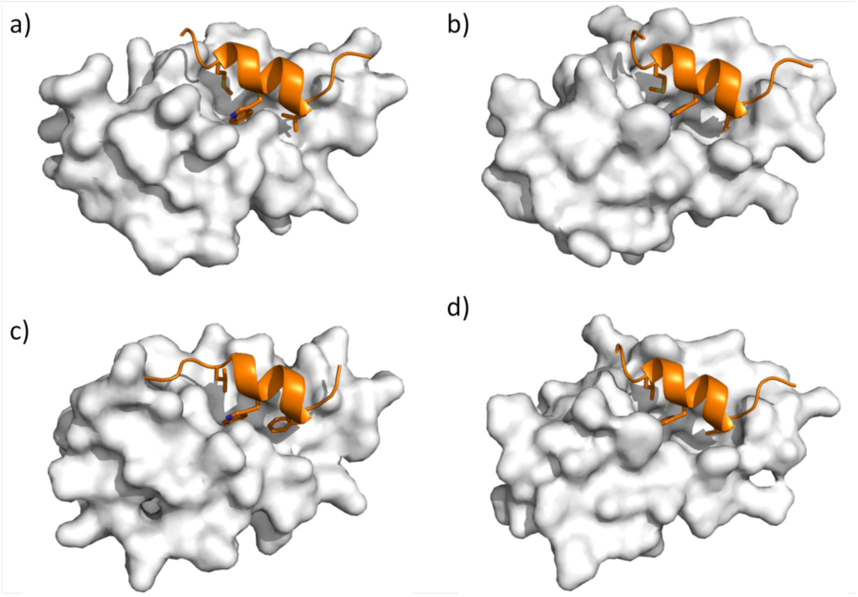

Figure S2: Homology models of (a) TaMdm2 bound to Tap53, (b) hMdm2 bound to Tap53, (c) TaMdm2 bound to hp53 and (d) hMdm2 bound to Tap53 M23L mutant. Mdm2 is represented as white surface while p53 peptide is shown in orange.

Figure S3 : Uncropped Western Blots.

Figure 2A:

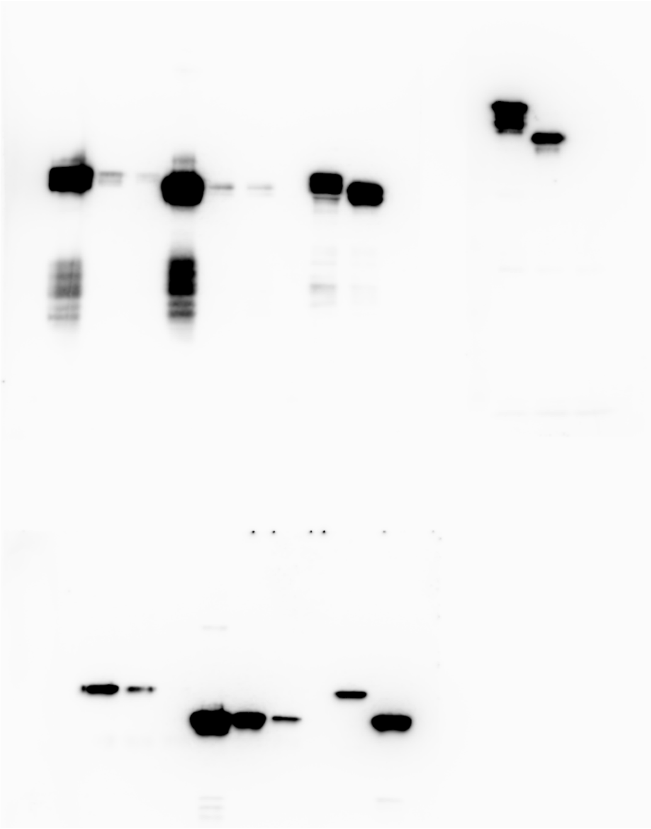

Figure 2B:

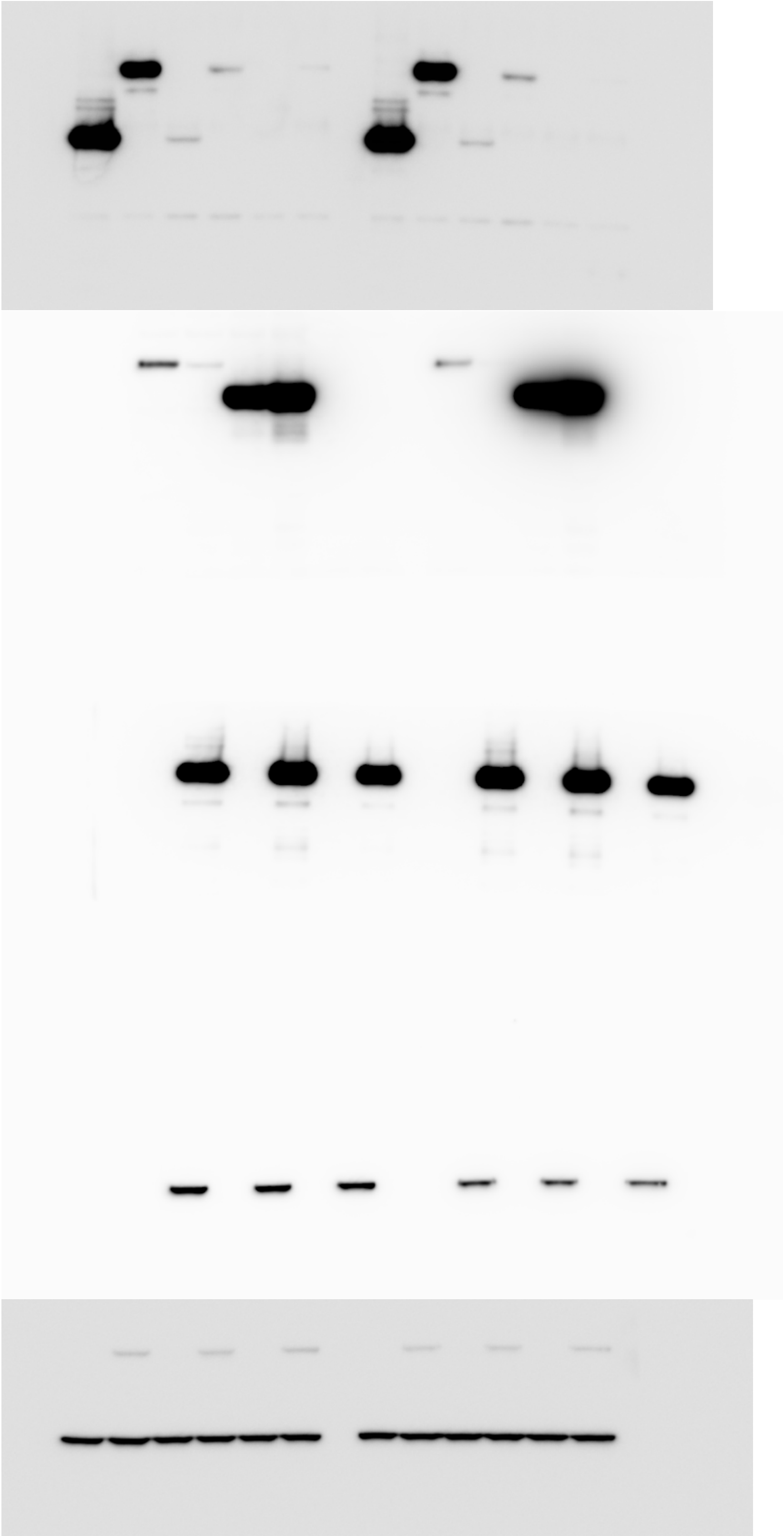

Figure 2C:

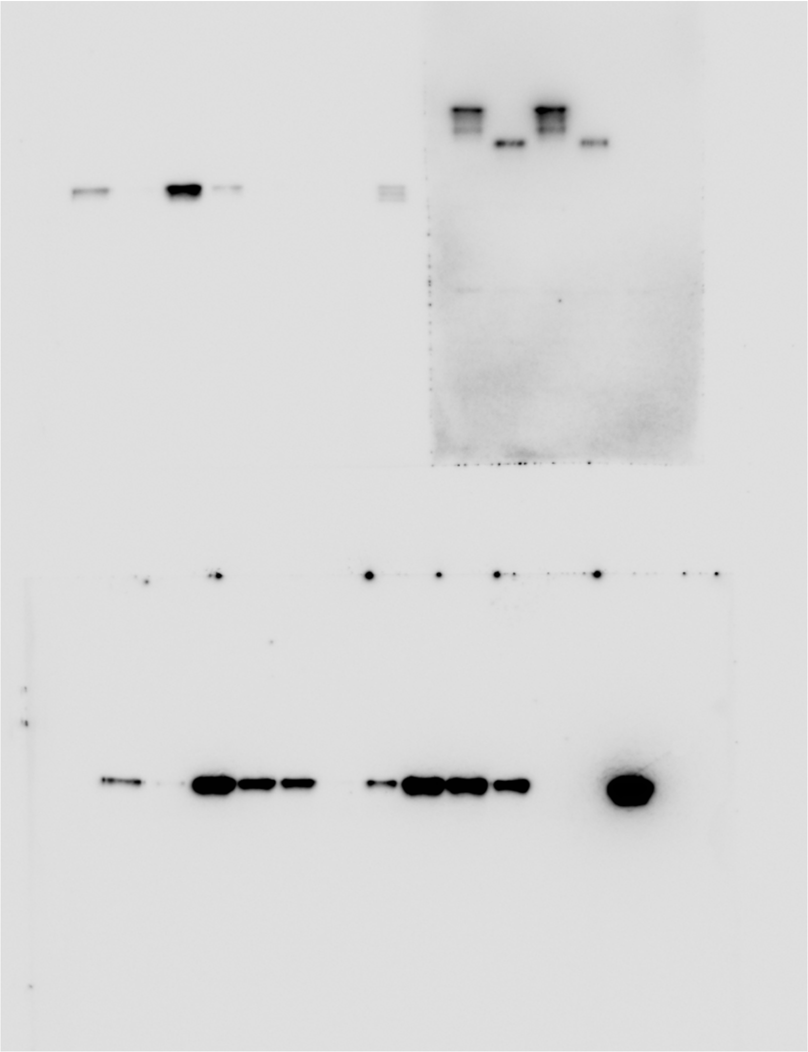

Figure 2D:

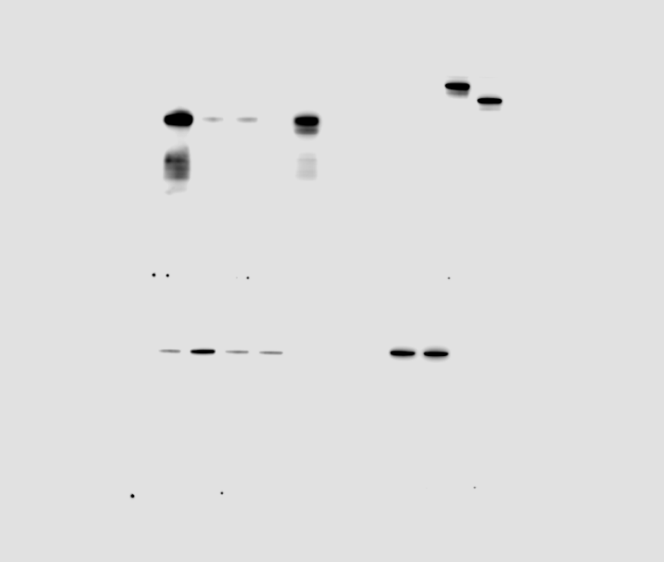

Figure 4C:

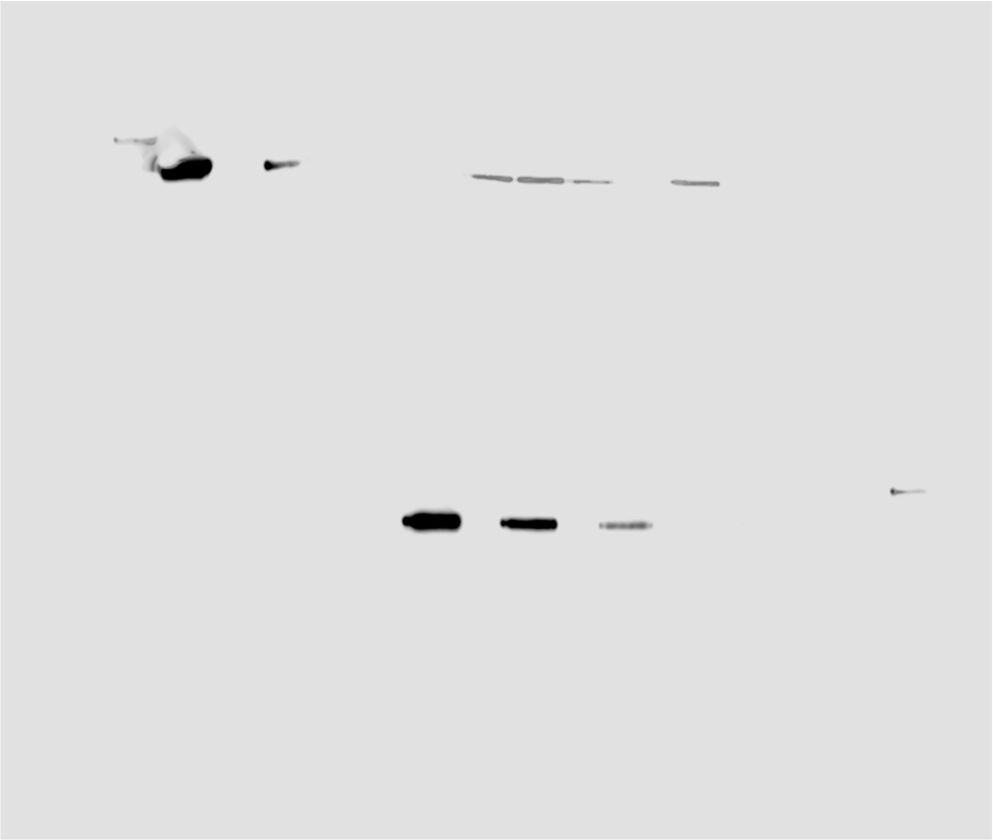

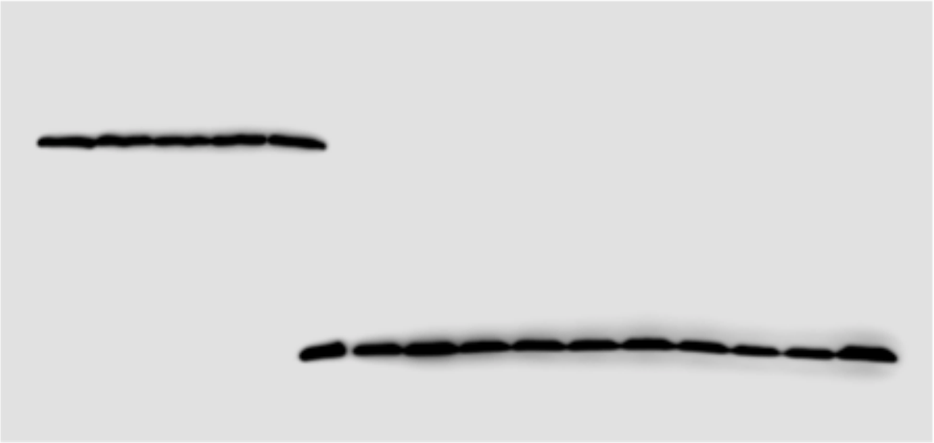

Figure 5:

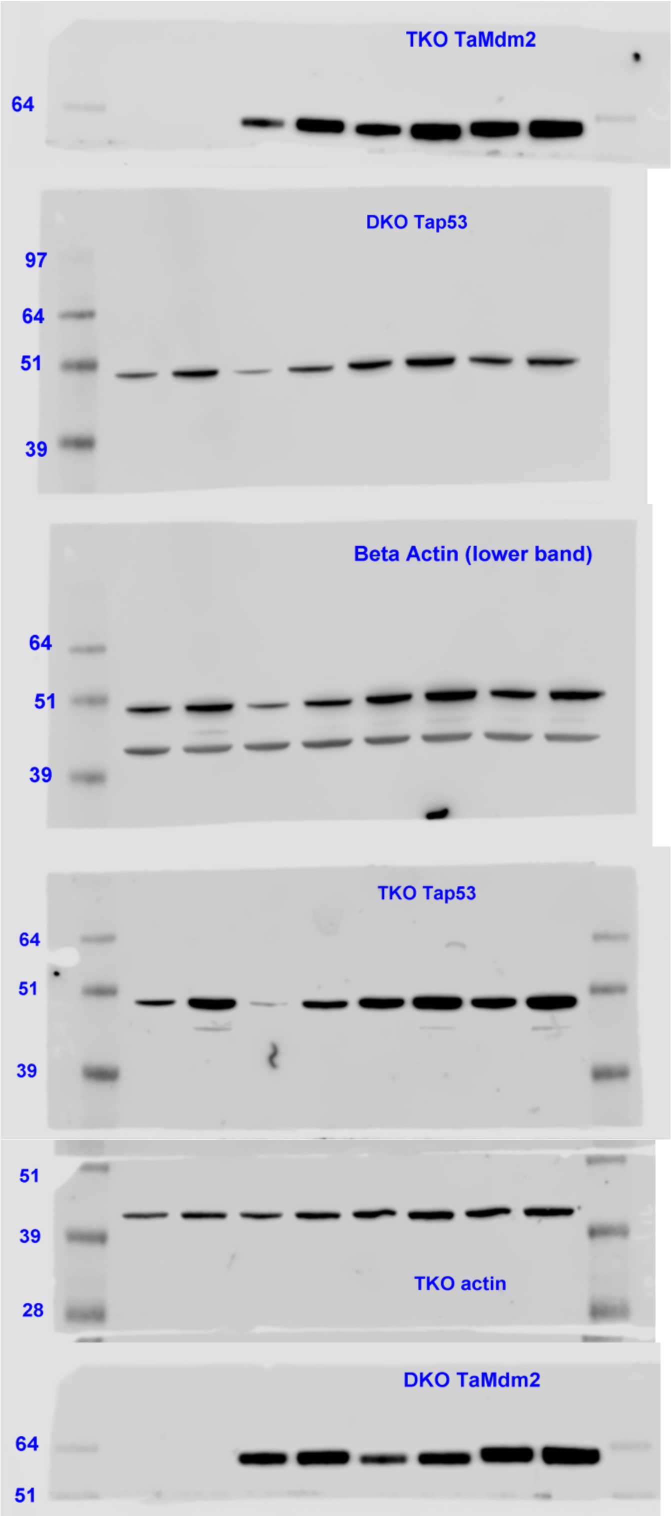

TKO TaMdm2
64
DKO Tap53
97
64
51
39
Beta Actin (lower band)
64
51
39
TKO Tap53
64
51
39
51
39
TKO actin
28
DKO TaMdm2
64
51

Figure 6A:

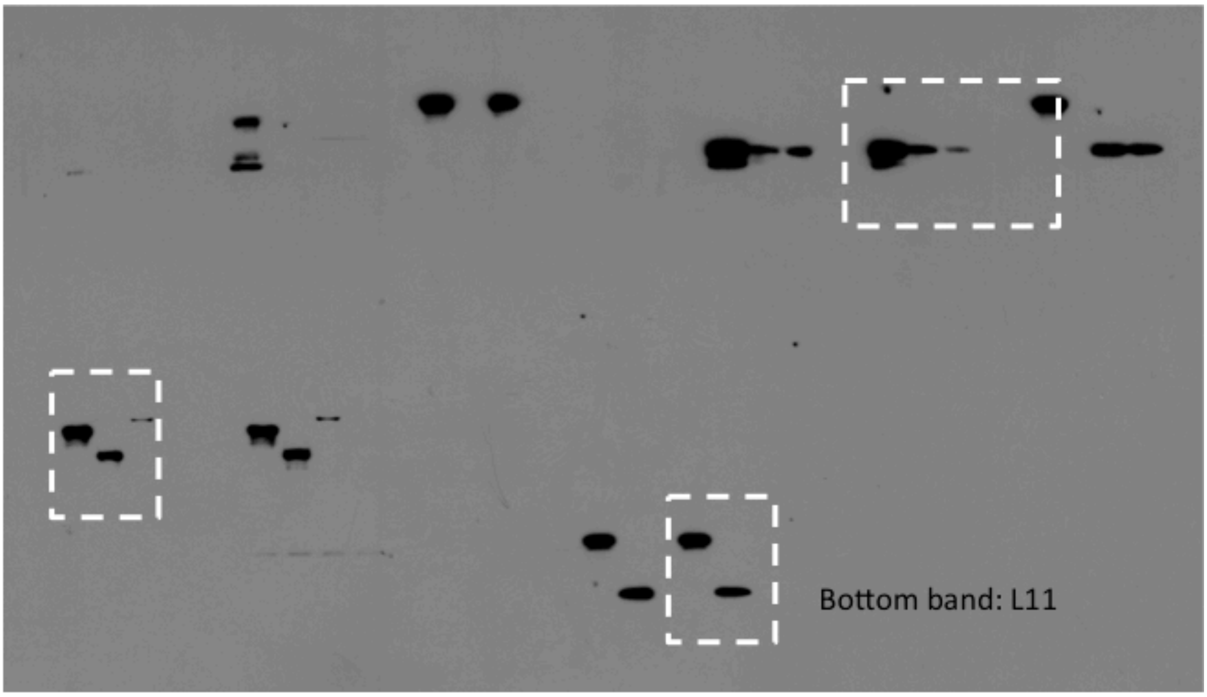

Figure 6B:

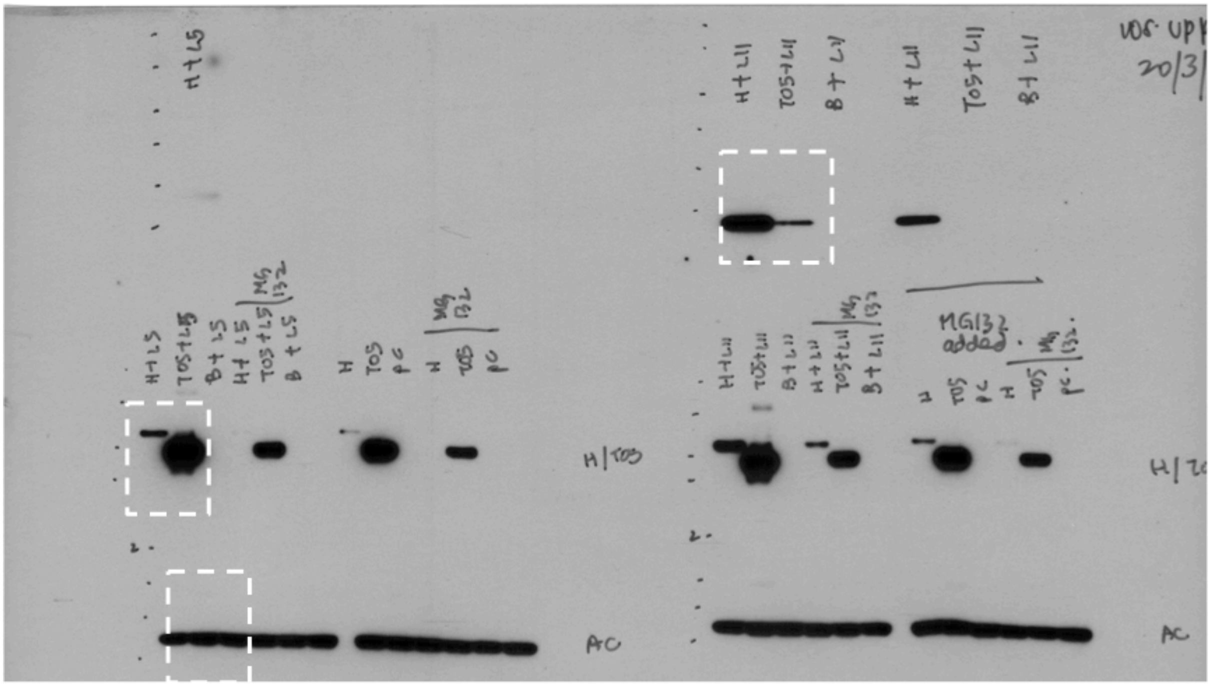

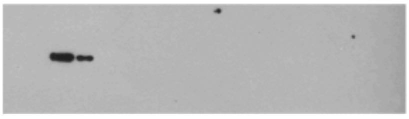

Figure S1:

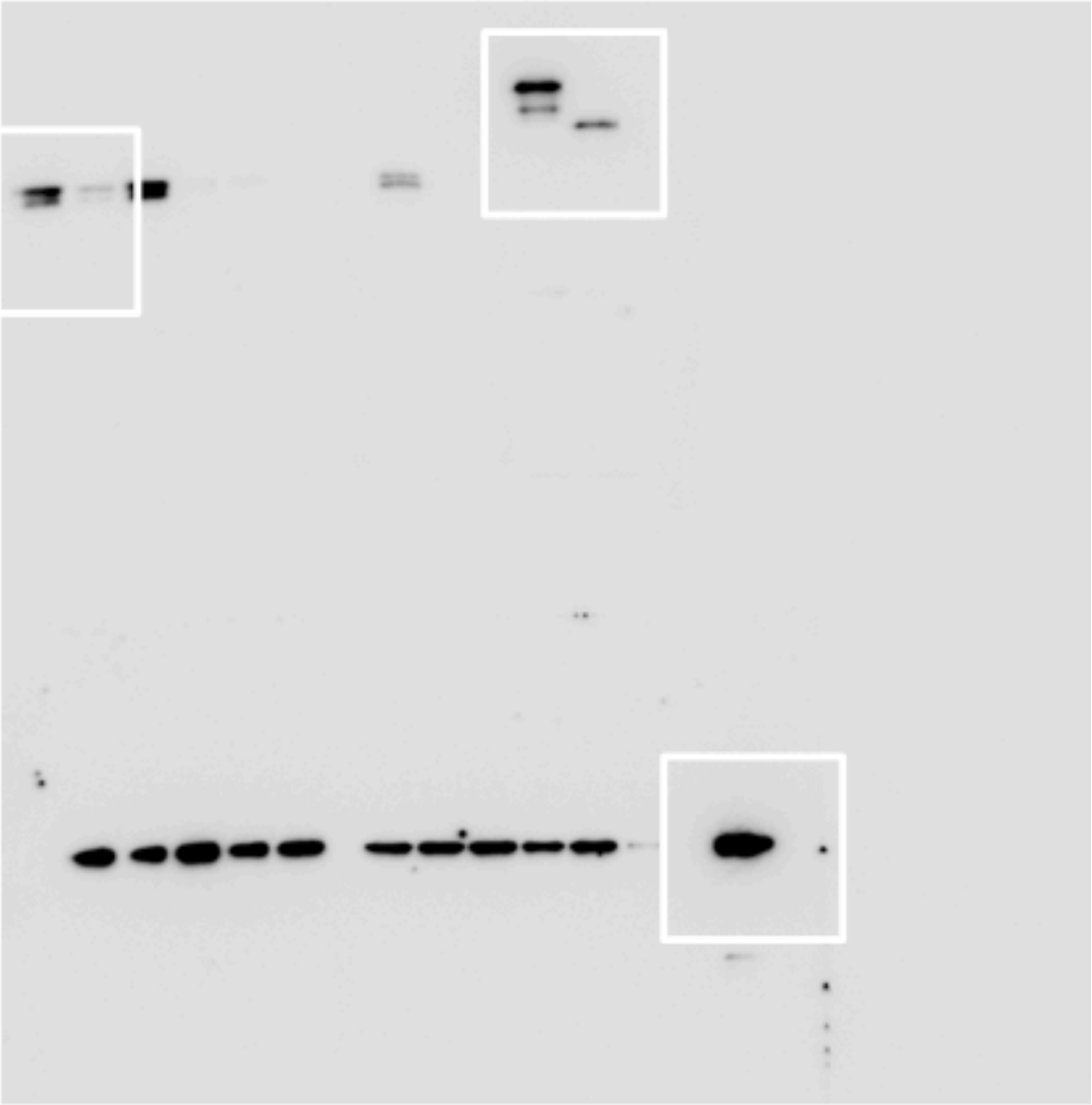

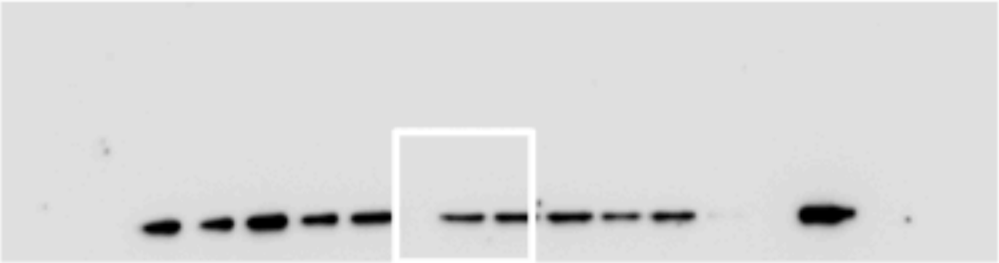

Supplementary Materials and Methods

Homology modelling

The amino acid sequences of Trichoplax p53 (Tap53) and Trichoplax Mdm2 (TaMdm2) were obtained from the UniProt database (accession numbers B3RZS6 and B3RT05 respectively) [1]. Template protein complex structures were obtained from the Protein Data Bank [2]. The structure of human Mdm2 (Mdm2) bound to a 12-mer peptide inhibitor (PDB code 3EQS) [3]) was used as a template to model the TaMdm2–Tap53 and Mdm2–Tap53 complexes, while the structure of the human Mdm2–Mdm4 RING domain heterodimer (PDB code 2VJF) [4]) was used as a template to model the TaMdm2 RING domain homodimer and TaMdm2–mMdm4 (mouse Mdm4) RING domain heterodimer. The 3EQS structure was chosen instead of the wild-type Mdm2–p53 complex as the Tap53 peptide is expected to have an additional α-helix turn due to the absence of a proline residue near its C-terminus. ClustalW [5] was used for sequence alignment. For each target structure, 10 multi-chain homology models were generated by MODELLER9v136 [6] and ranked by their DOPE score. The best model is the one with the lowest DOPE score and it was assessed for its stereochemical quality by PROCHECK [7]. Symmetry restraints were applied to build the model of the TaMdm2 RING domain homodimer.

References

1 Consortium, T. U. UniProt: a hub for protein information. *Nucleic Acids Res.* **43**, D204-D212 (2015).

2 Berman, H. M. *et al.* The Protein Data Bank. *Nucleic Acids Res.* **28**, 235-242 (2000).

3 Pazgier, M. *et al.* Structural basis for high-affinity peptide inhibition of p53 interactions with MDM2 and MDMX. *Proc. Natl. Acad. Sci. U. S. A.* **106**, 4665-4670 (2009).

4 Linke, K. *et al.* Structure of the MDM2/MDMX RING domain heterodimer reveals dimerization is required for their ubiquitylation in trans. *Cell Death Differ.* **15**, 841-848 (2008).

5 Thompson, J. D., Higgins, D. G. & Gibson, T. J. CLUSTAL W - improving the sensitivity of progressive multiple sequence alignment through sequence weighting, position-specific gap penalties and weight matrix choice. *Nucleic Acids Res.* **22**, 4673-4680 (1994).

6 Šali, A. & Blundell, T. L. Comparative protein modelling by satisfaction of spatial restraints. *J. Mol. Biol.* **234**, 779-815 (1993).
7 Laskowski, R. A., MacArthur, M. W., Moss, D. S. & Thornton, J. M. PROCHECK: a program to check the stereochemical quality of protein structures. *J. Appl. Crystallogr.* **26**, 283-291 (1993).
